# Comparison of the effectiveness of different home-based exercise programs in donors after liver transplantation: a randomized controlled trial

**DOI:** 10.1016/j.clinsp.2026.101094

**Published:** 2026-09-07

**Authors:** İlker Demir, Kezban Yigiter

**Affiliations:** a Department of Turgut Özal Medical Center Physical Therapy and Rehabilitation Unit, Inonu University, Malatya, Turkey; b Department of Physiotherapy and Rehabilitation, Hasan Kalyoncu University, Gaziantep, Turkey

**Keywords:** Liver transplantation, Donor, Exercise, Video based home exercise

## Abstract

**Background:**

The home exercise programme is a treatment method that ensures the continuity of rehabilitation after discharge. Although it is known that home exercise programmes improve recovery in patients compared to those who do not exercise at all in different diseases, it is not known which home exercise programme is more effective in donors after organ transplantation.

**Methods:**

A total of 117 donors were included in the study. The participants were randomly divided into three groups: control, home exercise, and video-based exercise groups. While no intervention was made to the control group, the individuals in the other groups were asked to perform the exercises twice a week for 8-weeks, for a total of 16 sessions. The evaluations were performed twice in the hospital environment, before and after the exercise.

**Results:**

The initial test results showed that the data for the groups were similar. In all measurements, a significant difference occurred between the first and second test scores in all three groups (p < 0.05). When comparing the changes in the groups, the brochure-based home exercise group showed a greater increase in the 6-minute walk test score compared to the control group, while the improvement rates in the video-based exercise group were superior to the other two groups in all data except for the non-dominant quadriceps femoris muscle strength (p < 0.05).

**Conclusion:**

Home exercise enhances recovery in donor rehabilitation after liver transplantation. When different home-based exercise programs were compared, the video-based exercise program was more effective than the standard brochure-based exercise program.

## Highlights

• Home-based exercise improved exercise capacity after liver transplantation.
• Exercise training reduced fatigue severity in liver transplant recipients.
• Home rehabilitation supported postoperative functional recovery.
• Supervised home exercises improved muscle strength outcomes.
• Home-based exercise programs may improve rehabilitation continuity.

## Introduction

Living donor liver transplantation is performed by implanting a piece of liver from the donor into the done. The high regeneration capacity of the liver is effective in preferring this transplantation procedure. The number of living donor liver transplantations is increasing thanks to advances in medical science and technology.^1^ With this increase, researching the health status of donors has become more important.

Liver transplantation is a major abdominal procedure for both the recipient and the donor. As a result, donors may experience symptoms after the operation that are similar to those observed after other major abdominal procedures. Incision pain, decreased functional capacity and physical activity levels,^2^^,^^3^ and increased fatigue are the main symptoms that donors may encounter after transplantation.^4^ Moreover, quality of life is negatively affected.^5^^,^^6^ Although these problems require the continuity of treatment of donors after discharge,^3^^,^^7^ the duration of hospital stay after transplantation makes medium and long-term follow-up and continuity of treatment difficult. In order to gain exercise habits and for the exercises to show their effectiveness, they need to be performed regularly for a certain period of time. However, donors are discharged and go to their homes after transplantation, which makes it difficult to do exercises with a physiotherapist in a hospital environment.

With the impact of technological developments in recent years, individuals can receive services from healthcare professionals without coming to the hospital environment using telecommunication technology. This development offers the chance for rehabilitation to individuals who have limited access to hospitals. Home-based exercise programs include different interventions, from telerehabilitation interventions that utilize telecommunication technology to programs that describe exercises with brochures. It was reported that home-based exercise practices produced positive results in different diseases.^8^^,^^9^ However, no study was found in the literature comparing the effects of home-based exercise programs on donors after liver transplantation.

In this study, the effects of different home-based exercise programs were compared in donors after liver transplantation in order to contribute to medium and long-term treatment options. To our knowledge, there is limited evidence regarding the comparative effectiveness of different home-based exercise programmes for living liver donors. The findings of this study could inform the development of structured post-discharge rehabilitation programmes for this group.

### Objective

The primary aim of the study was to develop new strategies for the continuation of post-discharge treatment programs for liver transplant donors.

Donors were evaluated in two different home exercise programs and in a control group that did not follow any exercise program.

Multiple physical parameters such as muscle strength, functional capacity, fatigue level, and dynamic balance were measured in the evaluation. This study addresses an area that has received relatively little attention and provides preliminary randomised evidence that could inform future research into donor rehabilitation.

## Materials and methods

The study was conducted at the Liver Transplantation Institute Hospital in Turgut Özal Medical Center affiliated to Malatya Inönü University between October 2024 and June 2025 to compare the effectiveness of different home-based exercise programs in donors after liver transplantation. During the study, the recommendations of the Consolidated Standards of Reporting Trials (CONSORT) group for conducting parallel group randomized trials were followed.^10^ The study was as a Clinical trial number: not applicable.

### Participants

The total number of participants was calculated as 117 donors with a power of 0.95. For this purpose, 152 donors were interviewed. 18 donors did not agree to participate, and 17 donors dropped out of the study. The study was completed with 39 people in each group and a total of 117 donors. Donors were randomly assigned to groups using a simple randomization method.

The study included liver transplant donors aged 18-years and over, volunteering to participate in the study, spontaneously breathing, hemodynamically stable, literate, without orthopedic disability or objectively identifiable functional capacity limitations that would prevent independent ambulation or participation in the exercise protocol (e.g., inability to complete the baseline 6-minute walk test independently, requirement for assistive walking devices, or documented musculoskeletal, neurological, or cardiopulmonary conditions restricting physical activity), conscious, and cooperative.

Those with cardiovascular, musculoskeletal, or neurological problems affecting physical mobility and walking and those using assistive devices were excluded from the study.

**Fig. 1:**
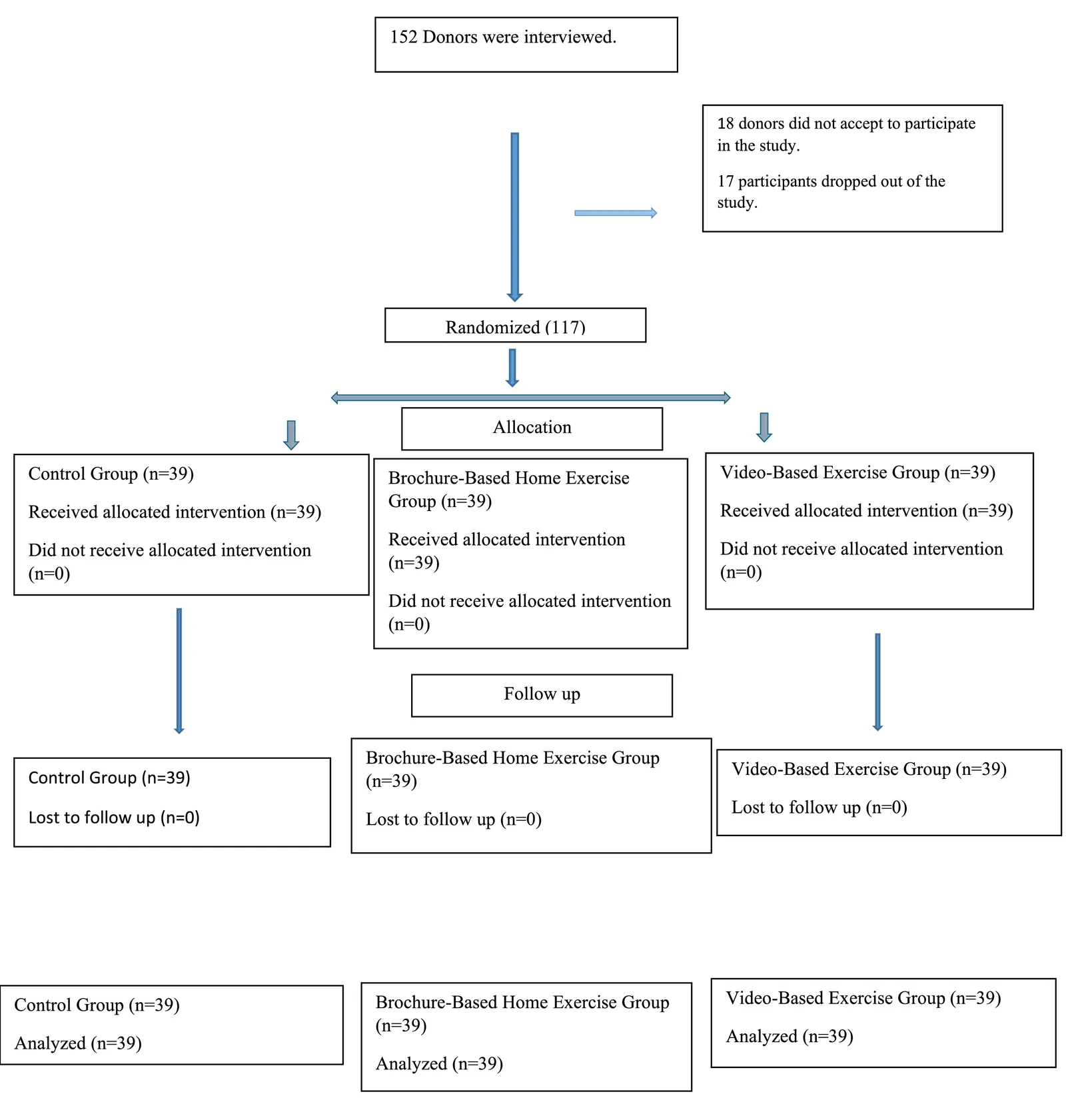
Study flow diagram.

The donors were divided into 3 groups by simple randomization method: control group (Group 1), standard brochure-based home exercise group (Group 2), and exercise group performed via video conference (Group 3). The study flow diagram is shown in Fig. 1.

### Exercise program

The exercise program was performed for 8-weeks, 2-days a week, and a total of 16-sessions. The exercises were taught to individuals in both groups in a hospital environment. A 7-day wait was required after the transplantation to ensure that the donors could mobilize on their own and that there was no restriction in their movements due to pain.

The individuals in the control group were not included in the exercise programs. At the end of the study, the donors in this group were allowed to participate in a program of their choice from a brochure-based home exercise program or a video-based exercise program. The participants in the home exercise group were given the exercises as brochures and were asked to do the exercises at home. The donors in the video-based exercise group performed the exercises with a physiotherapist via video calls on the WhatsApp program. WhatsApp was used solely for video calls, with no video or personal data recorded or stored.

The exercise programme consisted of warm-up exercises, isometric and isotonic contractions, and finally a cool-down programme. The exercises were performed at a level that the individual could tolerate, for 30‒45 min, with 8 repetitions in the first 4-weeks and 12 repetitions in the next 4-weeks, for a total of 8-weeks.

• Warm-up exercises were performed to include active joint range of motion exercises and shoulder circumduction movements. Participants performed active movements targeting the shoulder, elbow, hip, knee, and ankle joints within pain-free limits and at a controlled pace, with each movement performed for approximately 8 repetitions. The shoulder circumduction exercise was performed as part of this section; participants performed wide circular movements of the shoulder joint in a clockwise and counter-clockwise direction while keeping their bodies stable. All movements were planned to activate large muscle groups, and this section was performed to prepare the muscles for exercise, increase circulation, and support movement coordination.
• Isometric exercises: This exercise involved isometric contraction of the lower extremity extensor, adductor, and abductor muscle groups by applying pressure to a towel and consisted of a knee extension movement in a seated position. For the extensor muscle group, participants were asked to place a cylindrically folded towel under the distal part of their thighs and press down towards the floor to achieve isometric contraction of the quadriceps muscles. For the isometric contraction of the adductor muscle groups, a towel of the same shape was placed between the legs, and participants were asked to squeeze the towel with both legs. For the abductor muscle groups, the towel was placed on the outer side of the knee, and participants were asked to push their leg towards the wall. Each contraction was held for five seconds, followed by a five-second rest. Participants were asked to perform eight repetitions of the isometric contraction for the first four weeks and twelve repetitions for the following four weeks, provided there were no circumstances preventing them from performing the movement.
• The isotonic exercise programme consisted of standardized functional strengthening exercises targeting upper and lower limb muscle groups. The protocol included straight leg raises, hip flexion, mini squats, scapular retraction, sitting down and standing up from the bedside or chair.

The exercises were performed as described below: The straight leg raise exercise was performed in a supine position on a firm surface. The participant lay on their back with one knee bent, so that the foot was flat on the floor, while the leg being exercised was fully extended and the toes pointed upwards. After assuming the starting position, the straight leg was lifted 30‒45 degrees off the ground and held in this position for approximately 5 s before being lowered back to the starting position.

The hip flexion exercise was performed in a seated position with the trunk upright and the feet flat on the floor. Participants lifted one foot off the ground by flexing the hip and raising the knee toward the chest while keeping the trunk stable and avoiding compensatory movements. The position was held for 2–3 s and then the leg was lowered slowly back to the starting position. The movement was performed in a controlled manner for the prescribed number of repetitions and sets according to the exercise protocol.

The sitting-to-standing exercise performed at the edge of the bed or on a chair was implemented to improve lower extremity muscle strength, balance, and functional movement control. During the exercise, participants sat in a seated position, leaned their torso slightly forward, placed their feet flat on the floor, and stood up by extending their hips and knees; they then returned to the seated position in a controlled manner. Throughout the movement, inward collapse of the knees, lateral shifting of the torso, or sudden sitting movements were prohibited.

The mini squat exercise was performed standing, with feet shoulder-width apart and the torso upright. Participants were asked to squat in a controlled manner by pushing their hips back to bring their knees to approximately 30–45 degrees of flexion. During the movement, care was taken to ensure that the knees did not extend beyond the toes, that the knees did not buckle inwards, and that the torso did not lean forward excessively. After a brief pause in the lower position, participants used hip and knee extension to return to the starting position in a controlled manner.

The scapular retraction exercise was performed in a seated position. Participants were asked to pull their shoulder blades back and towards each other without moving their torsos. Care was taken to avoid unnecessary activation of the neck muscles during the movement and to maintain torso stability. When the scapulae were brought to the maximum retraction position, this position was held for a few seconds before returning to the starting position.

• Cool-down exercises were performed according to protocol, and this section included stretching exercises targeting the hamstrings, gastrocnemius, gastrosoleus, and pectoralis major and minor muscle groups. Stretching movements were performed in a controlled manner and within pain-free limits; each stretch position was held for approximately 15–30 s. This section was performed to support post-exercise muscle relaxation, regulate circulation, and reduce muscle tension.

### Data collection

Demographic information of the donors was recorded by asking about their age, marital status, employment status, body mass index, educational status, exercise habits, smoking and alcohol use.

The participants' functional capacities were measured with the 6-minute walking test, quadriceps muscle strength with the “make test”, dynamic balance with the timed up and go test, and fatigue levels with the fatigue severity scale.

• 6-Minute Walking Test (6MWT)

The test was performed according to the 6-MWT criteria references determined by the American Thoracic Society.^11^ The individuals were asked to walk as fast as they could, without running, down a straight 30-meter corridor for six minutes, and their distance was recorded.

• Muscle strength

Quadriceps femoris muscle test was measured with the “make test” technique, in which the individual applied maximum force towards the dynamometer in the physiotherapist’s hand for 5 s.^12^ The best measurement value, which was repeated three times at 30-second intervals on each side, was recorded in kilograms (kg).

• Timed up and go test

During the test, the time it took for the patient to get up from the chair s/he was sitting in, walk a distance of 3 m, then turn back and return to the starting position was measured.^13^

• Fatigue

The participants' fatigue level was measured using the fatigue severity scale consisting of 9 questions scored from 1 to 7.^14^

### Statistical analysis

IBM “Statistical Package for Social Sciences” (SPSS) statistical program was used to analyze the data. Sample size was determined by power analysis. Shapiro-Wilk and Kolmogorov-Simirnov tests were used to determine compliance with normal distribution. Quantitative data were summarized as mean ± standard deviation. The comparisons between the groups were examined using the ANOVA test with Bonferroni correction. Mixed-design Analyses of Variance (ANOVAs) were conducted for all outcome variables, with time (pre-test vs. post-test) as the within-subjects factor and group (control, brochure-based home exercise or video-based exercise) as the between-subjects factor. The primary effect of interest was the time × group interaction. Post hoc comparisons were performed using the Bonferroni adjustment where appropriate, and effect sizes were reported as partial eta squared (η²). A p-value of < 0.05 was considered statistically significant.

## Results

The mean age of the control group was 31.87±6.00 (min/max 21‒44) years, body mass index was 23.61±2.05 (min/max 19‒28) kg/m^2^ (kilogram/square centimeter); the mean age of the home exercise group was 34.33±8.96 (min/max 19‒50) years, body mass index was 24.56±2.91 (min/max 20‒29) kg/m^2^, and the mean age of the telerehabilitation group was 31.43±7.50 (min/max 20‒49) years, body mass index was 24.15±2.75 (min/max 19‒30) kg/m^2^.

Table 1. The proportion of male donors and married individuals was higher in all three groups. Although donors included secondary school and university graduates, the highest proportion of primary school graduates was seen in the brochure-based home exercise group. Although there were differences in demographic proportions between the groups, there was no statistically significant difference between them.

**Table 1:** Baseline demographic and clinical characteristics of the participants (n = 117).

Groups	Control Group		Brochure-Based Home Exercise Group		Video-Based Exercise Group	
Descriptive Characteristics	n	%	n	%	n	%
Gender						
Male	28	72%	30	77%	26	67%
Female	11	28%	9	23%	13	33%
Marital status						
Married	23	59%	26	67%	22	56%
Single	16	41%	13	33%	17	44%
Educational Status						
Secondary	15	38%	17	44%	15	38%
High School	17	44%	14	36%	14	36%
University	7	18%	8	20%	10	26%
Employment Status						
Working	16	41%	14	36%	16	41%
No-working	23	59%	25	64%	23	59%
Smoking Status						
Yes	17	44%	19	49%	17	44%
No	23	56%	20	51%	23	56%
Alcohol Use						
Yes	11	28%	9	23%	12	31%
No	28	72%	30	77%	27	69%
Exercise Habit						
Yes	9	23%	9	23%	7	18%
No	11	28%	14	36%	15	38%
Irregular	19	49%	16	41%	17	44%

Table 2. Based on pre- and post-measurement results from the eight-week program, donors showed improvement in exercise capacity, fatigue intensity, walking speed, and muscle strength. The video-based exercise group had the best second measurement results, while the control group showed the lowest scores.

**Table 2:** Pre-and post-exercise test scores.

Groups	Control Group		Brochure-Based Home Exercise Group		Video-Based Home Exercise Group	
	Pre-test	Post-test	Pre-test	Post-test	Pre-test	Post-test
6MWT (m)	434.51±47.98	476.05±45.27	432.69±46.55	482.35±41.00	425.69±50.09	515.17±51.97
Quadriceps Femoris (Dominant) Muscle Strength (kg)	10.51±3.46	13.69±3.91	10.25±3.40	15.33±3.27	9.76±2.84	17.46±3.33
Quadriceps Femoris (Non-Dominant) Muscle Strength (kg)	9.48±2.74	12.43±2.69	9.28±3.10	13.28±3.11	9.17±3.21	15.05±3.40
Timed Up and Go Test (*sec*)	8.00±1.05	6.56±0.82	7.87±0.97	6.07±0.89	8.02±0.87	5.94±0.79
Fatigue Severity Scale (score)	35.79±6.23	30.53±5.75	38.51±6.57	27.97±4.28	38.92±6.36	24.58±4.66

6MWT, Six Minute Walking Test; m, Meter; *sec*, Second; kg, Kilogram.

Table 3 shows Mixed-design analyses of variance were performed to examine the effects of time (pre-test vs. post-test), group (control group; brochure-based home exercise group; video-based exercise group) and the interaction between these factors on all outcome variables. The improvement rates in the groups were statistically significant between the first and second measurements, taken eight weeks apart (p < 0.05).

**Table 3:** Mixed-design ANOVA result for all outcome variables.

Outcome variables	Effect	df	F	p-value	Partial η²
6MWT (m)	Time (Pre vs. Post)	1, 114	24.90	<0.001	0.179
	Group	2, 114	2.63	0.076	0.044
	Time × Group	2, 114	35.37	<0.001	0.383
Quadriceps Femoris (Dominant) Muscle Strength (kg)	Time (Pre vs. Post)	1, 114	146.98	<0.001	0.563
	Group	2, 114	3.82	0.025	0.063
	Time × Group	2, 114	8.90	<0.001	0.135
Quadriceps Femoris (Non-Dominant) Muscle Strength (kg)	Time (Pre vs. Post)	1, 114	112.76	<0.001	0.497
	Group	2, 114	3.01	0.053	0.050
	Time × Group	2, 114	4.51	0.013	0.073
Timed Up and Go Test (*sec*)	Time (Pre vs. Post)	1, 114	292.54	<0.001	0.720
	Group	2, 114	2.32	0.103	0.039
	Time × Group	2, 114	3.22	0.044	0.053
Fatigue Severity Scale (score)	Time (Pre vs. Post)	1, 114	141.37	<0.001	0.554
	Group	2, 114	2.32	0.103	0.039
	Time × Group	2, 114	9.71	<0.001	0.146

6MWT, Six Minute Walking Test; m, Meter; *sec*, Second; kg, Kilogram, p < 0.05.

For the 6-Minute Walk Test, a significant main effect of time was observed (F(1, 114) = 24.90, p < 0.001, partial η² = 0.179), indicating an overall improvement in walking capacity. Furthermore, a significant time × group interaction was identified (F(2, 114) = 35.37, p < 0.001, partial η² = 0.383), demonstrating that the magnitude of improvement differed significantly between groups. The main effect of group was not statistically significant (p = 0.076). Quadriceps femoris muscle strength in the dominant leg showed a significant main effect of time (F(1, 114) = 146.98, p < 0.001, partial η² = 0.563) and a significant time × group interaction (F(2, 114) = 8.90, p < 0.001, partial η² = 0.135). This indicates that strength gains over time varied significantly between groups. A significant main effect of group was also observed (p = 0.025). Similarly, a significant main effect of time was found for non-dominant quadriceps femoris muscle strength (F(1, 114) = 112.76, p < 0.001, partial η²=0.497), together with a significant time × group interaction (F(2, 114) = 4.51, p = 0.013, partial η² = 0.073). However, the main effect of group did not reach statistical significance (p = 0.053). Analysis of Timed Up and Go test performance revealed a significant main effect of time (F(1, 114) = 292.54, p < 0.001, partial η² = 0.720), reflecting a substantial overall improvement in functional mobility. Additionally, a significant time × group interaction was observed (F(2, 114) = 3.22, p = 0.044, partial η² = 0.053), indicating differential changes between groups. However, the main effect of group was not significant (p = 0.103). For fatigue levels assessed by the Fatigue Severity Scale, a significant main effect of time was observed (F(1, 114) = 141.37, p < 0.001, partial η² = 0.554), indicating a reduction in fatigue over time. Notably, a significant time × group interaction was also detected (F(2, 114) = 9.71, p < 0.001, partial η² = 0.146), indicating that the reduction in fatigue differed significantly between groups. However, the main effect of group was not statistically significant (p = 0.103) (Table 3).

Table 4. One-way ANOVA revealed significant between-group differences in change scores for 6MWT (F(2114) = 35.37, p < 0.001), dominant quadriceps strength (F(2114) = 8.90, p < 0.001), non-dominant strength (F(2114) = 4.51, p = 0.013), fatigue severity (F(2114) = 9.71, p < 0.001), and timed performance (F(2114) = 3.22, p = 0.044). In the video-based exercise group and the control group, the improvement in the first and second test scores of muscle strength, dynamic balance, and fatigue severity was greater in the video-based exercise group (p < 0.05).

**Table 4:** Comparison of pre- and post-exercise test scores between the groups.

Outcome	F(2114)	η²	Video vs. Control	Video vs. Brochure	Brochure vs. Control
6MWT (m)	35.37	0.383	p < 0.001, MD = 131.02	p = 0.042, MD = 39.82	p < 0.001, MD = 91.20
Quadriceps Femoris (Dominant) Muscle Strength (kg)	8.90	0.135	p < 0.001, MD = 4.51	p = 0.049, MD = 2.61	p = 0.24, MD = 1.89
Quadriceps Femoris (Non-Dominant) Muscle Strength (kg)	4.51	0.073	p = 0.011, MD = 2.92	p = 0.18, MD = 1.87	p = 0.86, MD = 1.05
Timed Up and Go Test (*sec*)	3.22	0.053	p = 0.038, MD = −0.64	p = 0.80, MD = −0.28	p = 0.47, MD = −0.35
Fatigue Severity Scale (score)	9.71	0.146	p < 0.001, MD = −9.07	p = 0.036, MD = −5.28	p = 0.20, MD = −0.379

6MWT, Six Minute Walking Test; m, Meter; *sec*, Second; kg, Kilogram; MD, Difference Between Arithmetic Mean; η², Partial eta squared, Bonferonni Corrected ANOVA Test.

In the video-based exercise group and the brochure-based home exercise group, there was a statistically significant difference in dominant extremity Quadriceps femoris muscle strength and fatigue severity in favor of the video-based exercise group (p < 0.05).

**Fig. 2:**
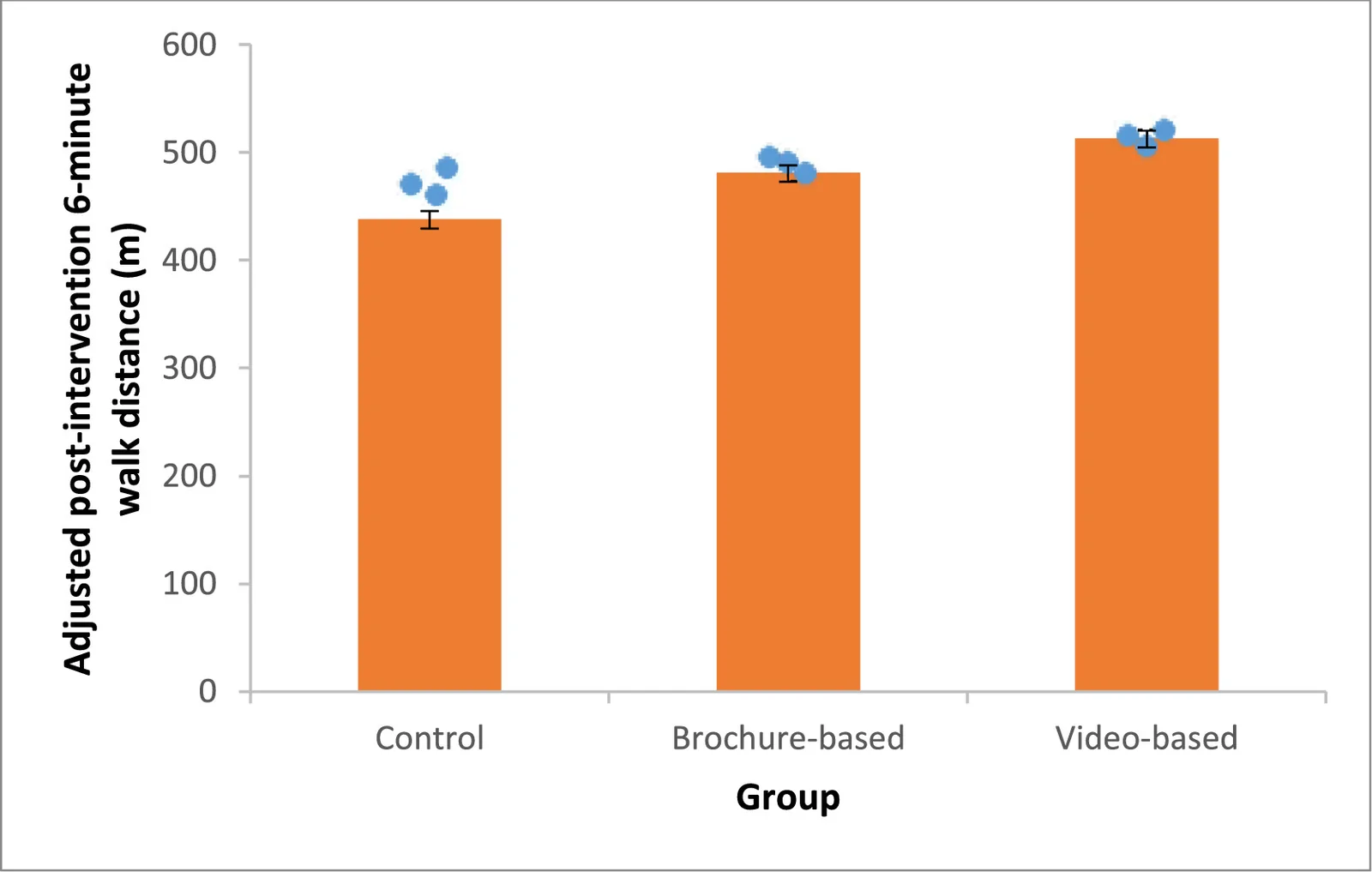
ANCOVA-adjusted mean changes in 6-minute walk distance across groups.

Fig. 2 shows the distribution of outcomes within each group, displaying the post-test values of individual participants. As baseline 6-Minute Walk Test (6MWT) performance differed between groups, an analysis of covariance (ANCOVA) was performed with baseline 6MWT entered as a covariate. After adjusting for baseline values, a significant main effect of group was observed in the post-intervention 6MWT distance (F(2, 113) = 20.71, p < 0.001). ANCOVA-adjusted post-intervention mean 6MWT distances were 438.0 ± 8.1 m for the control group, 481.0 ± 7.6 m for the brochure-based home exercise group and 513.0 ± 7.8 m for the video-based exercise group (see Fig. 2). Post hoc comparisons with Bonferroni correction indicated that the video-based exercise group achieved a significantly greater post-intervention walking distance than both the control group (p < 0.001) and the brochure-based group (p < 0.001). The brochure-based group also demonstrated significantly higher adjusted 6MWT values than the control group (p < 0.001).

## Discussion

Liver transplantation is a life-saving treatment option for end-stage liver disease. The rate of living donor liver transplantation has been increasing in recent years, making it more important to follow-up donors’ health. Post-transplant hospitalization periods make medium and long-term follow-up and treatment difficult. At this point, utilizing telecommunication technology may yield beneficial results. This randomized controlled trial compared the effectiveness of brochure- and video-based home exercise programmes for living liver donors during the early postoperative period. While improvements were observed in all groups, the video-based exercise group demonstrated significantly greater gains in functional capacity, quadriceps muscle strength and reduction in fatigue. These results imply that the way in which the exercises are delivered, rather than the exercises themselves, may substantially influence postoperative rehabilitation outcomes.

Our findings indicate that donors’ physical data are better after 8-weeks after transplantation. However, when this improvement was compared between the groups, it was seen that the video-based exercise group had better results than the standard brochure-based home exercise and control groups. The large effect sizes observed for the 6MWT (η² = 0.38) and fatigue severity (η² = 0.15) suggest that the differences between the different types of intervention may be clinically significant. However, these findings should be interpreted in light of the baseline differences and lack of assessor blinding. The superiority of the video-based exercise programme can be explained by physiological and behavioral mechanisms. From a behavioral perspective, Self-Determination Theory suggests that structured, interactive supervision can increase perceived competence through corrective feedback, foster relatedness through interaction with the therapist, and promote autonomy by enabling participation in the home environment. These factors are known to positively influence adherence and intrinsic motivation. Furthermore, behavioral accountability associated with scheduled live sessions may reduce non-adherence compared to unsupervised programmes. From a rehabilitation standpoint, real-time feedback is essential for motor learning and can optimize movement quality, intensity progression and neuromuscular activation. Additionally, supervised sessions may enhance perceived safety and exercise-related self-efficacy in patients recovering from major abdominal surgery. Together, these mechanisms may account for the greater improvements observed in functional capacity, muscle strength, and fatigue.

Although donors are healthy individuals, adverse effects are observed after the transplantation because the transplantation procedure is a major abdominal surgery. Compared to other types of transplantation, the rate of living donor liver transplantation has increased in recent years. This highlights the importance of structured postoperative follow-up strategies for donors, who are otherwise healthy individuals undergoing major abdominal surgery. However, since donors are healthy individuals, we believe that restoring their health after transplantation should be a priority. This study compared the effects of different home-based exercise programs in living liver donors, a population for whom comparative rehabilitation data remain limited. Therefore, we can only compare the effects of standard brochure-based home exercise and video-based home exercise interventions on recovery with the results in different diseases.

The mean age of the control group in the study was 31.87±6.00 years; the mean age of the home exercise group was 34.33±8.96 years; and the mean age of the telerehabilitation group was 31.43±7.50 years (min/max 20‒49). Sánchez-Cabús S et al.^16^ conducted a retrospective study on adult living liver donors with a mean age of 39-years. They found that 13% of donors developed minor complications and 8.7% developed major complications. Furthermore, the authors reported that the donors' average length of stay in the intensive care unit was 1-day and their average hospital stay was 7.5-days. Although living donors are typically young and healthy, these findings emphasize that liver donation is a significant surgical procedure with measurable postoperative morbidity. Therefore, structured rehabilitation strategies during the early post-discharge period are particularly important, even for relatively young donor populations, to help them recover functionally and reduce the potential impact of postoperative complications.

The 6MWT is a helpful, inexpensive, and equipment-free test used to assess exercise capacity before and after surgery, determining postoperative functional levels and postoperative pulmonary complications. However, walking tests performed in the early postoperative period after abdominal surgery can be challenging due to many factors, such as postoperative pain, drainage tubes, and orthostatic hypotension. In a study evaluating 6MWT results applied to predict the development of pulmonary complications after major surgery, it was emphasized that patients aged 60‒70 with a preoperative walking distance of <325 m had a higher risk of developing pulmonary complications.^17^ In our study, the 6MWT distance increased significantly more in the video-based exercise programme compared to the control group and brochure-based home exercise programmes. Although pulmonary complications were not assessed directly in our study, improved walking capacity may indicate enhanced cardiopulmonary endurance, functional mobility and faster postoperative recovery. Therefore, the greater improvement observed in the video-based group could indicate a more favorable functional recovery trajectory, which may be clinically relevant in minimizing postoperative risk in living donors. Further research incorporating objective outcomes of pulmonary complications is needed to determine whether improvements in function translate into a measurable reduction in postoperative morbidity.

No studies investigating the effects of home-based exercise programmes on extremity muscle strength in living liver donors were identified. Therefore, we made comparisons with studies conducted in other patient populations. Although telerehabilitation has been shown to have a positive effect on muscle strength and functional outcomes in orthopedic patients, such as those undergoing knee arthroplasty, these findings must be extrapolated to living liver donors with caution. Unlike patients with chronic musculoskeletal conditions, donors are individuals who were previously healthy and are now recovering from major abdominal surgery. Therefore, their rehabilitation responses and adherence patterns may differ substantially.^15^ Lim JY et al. compared the effects of brochure-based home exercise with telerehabilitation-based home exercise interventions on quadriceps muscle strength after knee arthroplasty. At the end of the study, it was reported that although an increase was observed in the groups in the 6th, 12th, and 24th weeks, there was no significant difference between the groups.^18^ Studies investigating home-based exercise programmes following knee arthroplasty have produced inconsistent results, which is likely due to variations in supervision intensity, technological support and patient characteristics. In our study, all groups demonstrated improvements in muscle strength over time. However, the video-based exercise group showed significantly greater gains than the other groups. This difference may be due to the benefits of real-time supervision, immediate corrective feedback and increased adherence that come with synchronous interaction. Furthermore, unlike orthopedic patients, who often begin rehabilitation with pre-existing muscle weakness or joint pathology, living liver donors may respond more quickly to structured neuromuscular activation, given their relatively preserved baseline physical capacity. These factors may partly explain why a significant difference between groups was observed in our study, in contrast to some findings in orthopedic populations.

The effects of different home-based exercise programs have not been investigated only in orthopedic patients. Telerehabilitation and brochure-based home exercise program for 8-weeks were applied to patients with systemic sclerosis and pre- and post- exercise measurements were made. It was reported that fatigue, pain, and depression levels decreased and the quality of life of the patients increased in both groups, but the telerehabilitation group was superior to the standard home exercise program.^19^ Although pulmonary rehabilitation is known to be beneficial in patients with chronic obstructive pulmonary disease, many patients may not be able to access pulmonary rehabilitation or continue it for a long-term. For this purpose, telerehabilitation and unsupervised home exercise interventions were applied to the patients and the results were examined. At the end of the study, it was reported that both methods reduced hospitalizations.^20^ From a clinical perspective, these findings suggest that structured, supervised, home-based exercise programmes could be beneficial for populations where continuous in-person rehabilitation is difficult to achieve. For living liver donors, who are discharged early and may not have access to long-term supervised rehabilitation, video-based supervision could be a useful way to improve adherence, ensure exercise safety and optimise functional recovery. Therefore, implementing interactive home-based rehabilitation programmes in transplant centres could help to bridge the gap between early discharge and full recovery. Video-based home exercise programmes could be particularly beneficial in transplant centres with limited rehabilitation resources, or where patients encounter geographical barriers. Incorporating structured remote supervision into standard postoperative follow-up protocols could enhance recovery without increasing the burden on hospitals.

Studies suggest that home-based exercise programs contribute to recovery. However, the results differ in terms of the superiority of telerehabilitation and unsupervised exercise programs. Many factors such as the type and stage of the disease, the exercise program, and the duration of the program may be effective in this separation. Our study found that brochure-based home exercise interventions effectively improved the functional capacity, muscle strength, balance and fatigue of living liver donors. However, the video-based home exercise group showed greater improvements. From a clinical perspective, this finding suggests that adding real-time supervision to similar exercise protocols could enhance their effectiveness. For populations such as living liver donors, who are discharged early and may lack structured follow-up, interactive supervision could optimize exercise performance, adherence and confidence during recovery. Therefore, even when the content of the exercises is similar, the way in which they are delivered may significantly influence rehabilitation outcomes.

Several limitations of this study should be acknowledged. The limitations of the study include the fact that the study was conducted in a single center and that it is not possible to compare this study with another study in literature. Another limitation is that although participants in the control group did not receive a structured exercise intervention, they were not informed about avoiding physical activity during the follow-up period. Therefore, it cannot be excluded that control participants engaged in unsupervised or self-directed exercise. This may have resulted in partial contamination of the control group, which could have reduced the observed differences between the groups. Another limitation is that due to the practical constraints of the intervention design, outcome assessors were not blinded to group allocation. The same physiotherapist supervised the exercise sessions and conducted the outcome assessments, which may have introduced detection bias. However, standardized assessment procedures were used for all groups to minimize potential measurement bias. Future studies should consider monitoring physical activity levels in all groups or providing clearer instructions on exercise behavior during the study period. We believe that studies in this area will contribute to the recovery of donors who do not have the opportunity for long-term treatment in a health center after liver transplantation.

Our findings suggest that the video-based home exercise program is superior to the control and standard brochure-based home exercise programs. Our findings suggest that structured, interactive home-based exercise programs may enhance postoperative recovery in living liver donors compared to unsupervised formats. This study adds to the limited body of evidence regarding structured home-based rehabilitation strategies in living liver donors and may inform future program development in settings where continuity of in-person treatment is challenging.

## Ethic statement

Prior to data collection, the necessary permission was obtained from the Liver Transplantation Institute of Inonu University and ethical approval was obtained from the Non-Interventional Researchs Ethics Committee of Hasan Kalyoncu University (Decision n° 2024/117, Date: 15.10.2024). Furthermore, all individuals were informed about the study and those who voluntarily agreed to participate were included. They were also informed that their information would be protected and that they could withdraw from the study upon request. In the video-based exercise group, real-time supervision was provided via WhatsApp video calls, which were used solely for live visual communication during exercise sessions. No video or audio recordings, screenshots or personal data were recorded, stored or shared via the app. All study data were anonymised and stored securely. No personal identifiers were processed or stored via third-party applications. The research was conducted in accordance with the principles of the Declaration of Helsinki.

## Consent for publication

Written informed consent was obtained from all participants for the publication of their personal or clinical details and any accompanying images in this study.

## Authors’ contributions

İlker Demir: Writing-review & editing; Writing-original draft; Methodology; Investigation; Formal analysis; Data curation. Kezban Yigiter: Writing-review & editing; Writing-original draft; Methodology.

## Funding

This research received no financial support from any organization.

## Data availability statement

The datasets generated and/or analysed during the current study are not publicly available due to patient privacy and ethical restrictions but are available from the corresponding author on reasonable request.

## Declaration of competing interest

The authors declared no potential conflicts of interest with respect to the research, authorship and/or publication of this article.
